# Comment on “Correlation Analysis of 24‐h Minimum Heart Rate Levels and Deceleration Capacity of Heart Rate in Patients With Type 2 Diabetes Mellitus Complicated With Essential Hypertension”

**DOI:** 10.1111/anec.70205

**Published:** 2026-06-08

**Authors:** Mawra Naveed, Mashal Naveed, Intzar Ahmed, Muhammad Sajjad, Mahnoor Jan

**Affiliations:** ^1^ Shaikh Khalifa Bin Zayed Al Nahyan Medical and Dental College Lahore Pakistan; ^2^ Allama Iqbal Medical College Lahore Pakistan; ^3^ Islam Medical College Sialkot Pakistan

To the Editor,

We read the article titled “Correlation Analysis of 24‐h Minimum Heart Rate Levels and Deceleration Capacity of Heart Rate in Patients With Type 2 Diabetes Mellitus Complicated With Essential Hypertension” by Wu et al. with great interest (Wu et al. 2026). In this study, the authors add to the non‐invasive assessment of cardiovascular risk in a high‐risk population by examining the deceleration capacity (DC) and the 24‐h minimum heart rate (MHR). However, despite its clinical relevance, several limitations merit further attention.

First, as this study is cross‐sectional, the cause and effect relationship between the variables is constrained. Although the study explained the relationship between MHR and DC, it failed to follow patients over time; therefore, it is not possible to determine which variable influences the other. However, Bauer et al. (2006) proved in their study that the real value of DC lies in its capability to predict future outcomes, which is only meaningful in case patients are followed over time. Thus, in order to better understand this relationship, further studies should concentrate on longer follow‐up and use longitudinal study designs.

Second, this study fails to account for glycemic control (HbA1c levels), duration of diabetes, and severity of diabetic autonomic neuropathy. As Vinik et al. (2003) clearly explained in their study, poor glycemic control is strongly linked to the advancement of cardiac autonomic neuropathy. Since diabetes‐related factors were not taken into consideration, it cannot be determined whether the reduction in DC is truly due to MHR or simply a result of more severe diabetes. Therefore, future studies should include metabolic parameters to get clearer and more reliable results.

The third major limitation of this study is the absence of evaluation of clinical cardiovascular outcomes such as arrhythmias, hospitalization, or mortality. Without outcome‐based follow‐up, the clinical significance of altered MHR and DC remains uncertain. Bauer et al. showed that impaired DC predicts adverse cardiovascular outcomes following myocardial infarction (Bauer et al. 2006). Future studies should include follow‐up data with defined cardiovascular endpoints.

Lastly, the failure to account for circadian rhythm and sleep parameters when measuring minimum heart rate (MHR) was another significant limitation of this study. Although Holter monitors allowed for continuous heart rate tracking, this investigation did not account for different stages of sleep nor autonomic shifts throughout sleep. Because MHR typically occurs during sleep and is reflective of parasympathetic (vagal) tone, these physiological differences were not accounted for when assessing overall autonomic function. Trinder et al. (2001) have demonstrated autonomic modulation differs across sleep stages. Thus, future investigations should use polysomnography or actigraphy in order to assess more specific and circadian‐adjusted heart rate dynamics.

In conclusion, this study highlights a possible relationship between MHR and DC in patients with type 2 diabetes mellitus and essential hypertension. However, a few limitations discussed earlier may have influenced the findings. Future research should address these limitations by adopting prospective longitudinal designs, including assessments of glycemic control and autonomic neuropathy, incorporating cardiovascular outcome follow‐up, and considering circadian and sleep‐related variations to enhance the validity and clinical relevance of the findings.

## Author Contributions

All authors meet the ICMJE authorship criteria. All of the authors contributed to the planning, writing, revision, and final approval of the manuscript. All authors agree to be accountable for all aspects of the work and ensure the accuracy and integrity of the manuscript.

## Funding

The authors have nothing to report.

## Disclosure

Transparency statement: The Authors affirm that this manuscript is an honest, accurate, and transparent account of the study being reported, that no important aspects of the study have been omitted, and that any discrepancies from the study as planned (and if relevant, registered) have been explained.

## Ethics Statement

The authors have nothing to report.

## Consent

The authors have nothing to report.

## Conflicts of Interest

The authors declare no conflicts of interest.

## Data Availability

Data sharing does not apply to this article as no datasets were generated during the current study; all data were sourced from published literature.

## References

[anec70205-bib-0001] Bauer, A. , J. W. Kantelhardt , P. Barthel , et al. 2006. “Deceleration Capacity of Heart Rate as a Predictor of Mortality After Myocardial Infarction: Cohort Study.” Lancet 367: 1674–1681. 10.1016/S0140-6736(06)68735-7. 16714188

[anec70205-bib-0002] Trinder, J. , J. Kleiman , M. Carrington , et al. 2001. “Autonomic Activity During Human Sleep as a Function of Time and Sleep Stage.” Journal of Sleep Research 10: 253–264. 10.1046/j.1365-2869.2001.00263.x. 11903855

[anec70205-bib-0003] Vinik, A. I. , R. E. Maser , B. D. Mitchell , and R. Freeman . 2003. “Diabetic Autonomic Neuropathy.” Diabetes Care 26: 1553–1579. 10.2337/diacare.26.5.1553. 12716821

[anec70205-bib-0004] Wu, P. , Z. Xu , X. Zheng , et al. 2026. “Correlation Analysis of 24‐h Minimum Heart Rate Levels and Deceleration Capacity of Heart Rate in Patients With Type 2 Diabetes Mellitus Complicated With Essential Hypertension.” Annals of Noninvasive Electrocardiology 31: e70189. 10.1111/anec.70189. 41987427 PMC13084145

